# Servant Plague

**Published:** 1879-10

**Authors:** 


THE utterly trifling character of many of our cooks, nurses, and housemaids, is one of the chief ingredients of domestic inquietude. It makes more mad women, more grumpy men, and keeps up more incessant annoyance in a family, than all other causes combined. It worries the wife, saddens the husband, breaks in upon the enjoyment of the children, and keeps multitudes of houses in a state of irritation and unrest, which eats out half the enjoyment of domestic life. No wonder the disappointed husband flies to the club-house, the billiard table, the whist party, or the "saloon" of gilded guilt, where external gorgeousness pleases the eye, and delusive drinks steal away the senses—while the high priests of the faro-bank and the card-pack steal the purse , leaving nothing to tell the tale but the bloated face, the bankrupt counter, and the blasted reputation. No wonder that young girls stray away to the ball-room, and young boys to the theatre or the negro opera. No wonder that the overtaxed wife fails, and fades, and pales away,—or, mounting a mettled nag, rows her husband up salt river!

Let then, all independent housekeepers make a strike for higher compensation for the monthly seven dollars paid the nurse or chamber-maid, and the eight dollar cook, who can eat "pastry" but can't make it, who expects you to get all your bread at the baker's, and "stipulates" that your washing is to be hired out; who gives you to understand that she must have loaf sugar in her tea and coffee, as "brown" gives her the headache; who must have green tea for breakfast; that the front gate must not be kept locked, as it looks as if you didn't want any of her friends to come and see her; whose "perquisites" are all the "soap fat," for which she gets two cents a pound, made up of fresh lard costing sixteen cents, and butter at thirty-four cents, with an occasional slice from the sixteen cent ham, and the fourteen cent fresh pork—it being "understood" that besides the weekly afternoon out, which extends from three o'clock P. M. to midnight, she is to have a chance at church on Sunday, and the occasional christenings and funerals, averaging, as every housekeeper may conjecture, once a week, on which occurrences you need not expect to see them until next day after breakfast, stupefied with want of sleep, worn out with the all-night carousal, and far more fit for a bed in a hospital than for the duties of a house servant.

